# Sources of Oxidative Stress in Parkinson’s Disease: Pathways and Therapeutic Implications

**DOI:** 10.3390/antiox15020187

**Published:** 2026-02-02

**Authors:** Yordan Yordanov, Denitsa Stefanova, Magdalena Kondeva-Burdina, Virginia Tzankova

**Affiliations:** Department of Pharmacology, Pharmacotherapy and Toxicology, Faculty of Pharmacy, Medical University of Sofia, Dunav 2 Str., 1000 Sofia, Bulgaria; denitsa.stefanova@pharmfac.mu-sofia.bg (D.S.); mkondeva@pharmfac.mu-sofia.bg (M.K.-B.); vtzankova@pharmfac.mu-sofia.bg (V.T.)

**Keywords:** Parkinson’s disease, etiology, personalized therapy, disease modifying, novel pharmacotherapy

## Abstract

Parkinson’s disease (PD) is a heterogeneous neurodegenerative disorder in which oxidative stress represents a final common pathway linking diverse genetic and environmental insults to dopaminergic neuron loss. This review synthesizes evidence on how the commonly observed pathological changes in PD converge on excessive reactive oxygen species generation and redox imbalance. We present an overview on these pathways and key PD-linked genes that perturb mitochondrial quality control, lysosomal function, and inflammatory signaling, reinforcing oxidative stress. The major classes of redox-targeted therapeutic strategies under preclinical and clinical evaluation are outlined. Although many candidates show robust target engagement and neuroprotection in models, clinical trials have frequently yielded neutral or modest results, highlighting challenges related to brain delivery, off-target effects, optimal treatment window, and the fact that oxidative stress alone may be necessary but not sufficient to drive human disease progression. In the current paper, beyond cataloguing oxidative pathways, we explain the role of etiologic heterogeneity on biochemical target engagement and clinical outcomes. We outline subtype-enriched trial strategies and rational combination approaches. Targeting oxidative stress–related pathways thus remains a promising avenue for disease modification in PD, provided that future interventions are mechanistically informed and adapted to patient-specific redox vulnerabilities.

## 1. Introduction

Parkinson’s disease (PD) is a common age-associated neurodegenerative disorder pathologically defined by progressive loss of substantia nigra (SN) pars compacta dopaminergic neurons and the typical accumulation of α-synuclein-rich Lewy bodies [1]. Up to now, therapies have provided symptomatic relief and limited evidence of disease-modifying effects for some approved drugs [2].

While the precise etiopathogenesis remains complex and multifactorial, converging evidence implicates oxidative stress as a common critical amplifier and downstream driver of SN neurodegeneration [3,4]. Oxidative stress occurs when generation of reactive oxygen (ROS) and reactive nitrogen species (RNS) predominates over antioxidant defenses, leading to damage of cellular lipids, proteins, and nucleic acids [5].

The SN is especially vulnerable to oxidative damage due to dysfunctional metabolic turnover and adenosine triphosphate (ATP) requirements [6], dopamine oxidation, high iron content [7], and relatively lower antioxidant capacity [8]. Indeed, post-mortem and biochemical laboratory studies in PD patients consistently demonstrate elevated markers of systemic oxidative damage compared to age-matched controls [9] and impaired antioxidant status [10].

Multiple pathogenic factors in PD either stem from or exacerbate oxidative stress. Mitochondrial dysfunction, particularly complex I deficits in the electron transport chain, is a well-documented feature in PD and a potent source of ROS [11]. It can be caused or exacerbated by dysfunctions in diverse cellular processes. These observations make a compelling case that affecting “druggable” targets, functionally related to oxidative stress, could have disease modifying effects in PD [4]. The PD Map [12] is a comprehensive curated source of such pathways, related to PD etiology.

Intensive research is being carried out to refine antioxidant therapeutic strategies, from repurposing safe, brain-penetrant compounds, e.g., antidiabetes drugs and dietary supplements, to developing novel agents targeting redox-sensitive proteins or transcriptional networks. Some of the experimental approaches include bolstering endogenous antioxidant defenses, for example, activating the Nrf2 pathway, central for supplementing endogenous antioxidant systems. Others reduce the production of ROS by inhibiting enzymes like monoamine oxidase-B or chelating redox-active iron. A number of candidate drugs have progressed to clinical trials [13,14]. Some have shown biochemical efficacy, such as mitochondria-targeted drugs [15] that raise glutathione (GSH) or dicotinamide adenine dinucleotide (NAD^+^) levels in the central nervous system (CNS) [16] or iron chelation [17], or lower brain iron content [17,18]. Several phase II/III trials targeting such pathways have recently yielded negative or inconclusive results [19].

Yet, translating biochemical changes into slowed neurodegeneration or improved clinical outcomes has proven difficult. Despite the challenges, the rationale for countering oxidative stress in PD remains strong, as it is one of the earliest and most ubiquitous changes detected in PD pathology and not merely a consequence of neuron death [17,18]. Moreover, individuals with higher systemic antioxidant capacity, e.g., higher urate levels, have shown slower PD progression in epidemiological studies [19].

The current paper provides, beyond merely cataloging oxidative pathways, an integrated overview of the mechanisms by which oxidative stress contributes to PD at the molecular level, the relationship with current therapeutic approaches, and potentially effective novel strategies to modulate these pathways, pointing to personalized strategies to effectively translate these into disease-modifying treatments.

## 2. Etiological Mechanisms of PD

**Figure 1:**
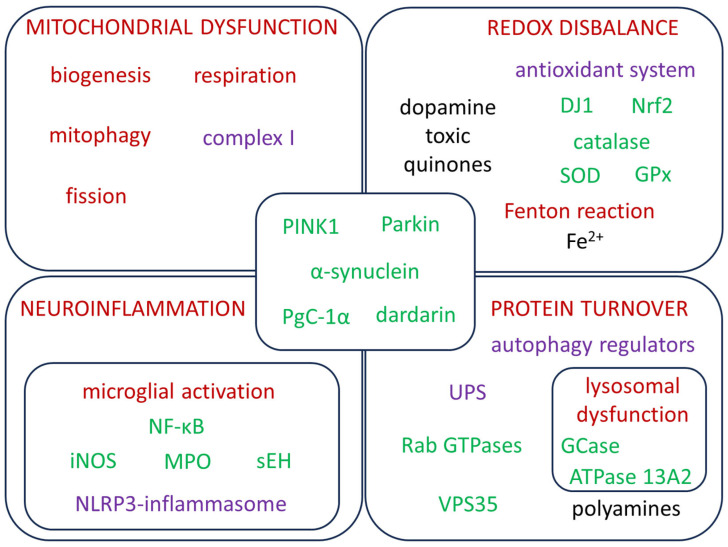
Major PD-related biological processes (dark red), functional complexes (purple), and proteins (green), intertwined with oxidative stress pathways, the dysfunction of which results in oxidative damage. Abbreviations: DJ1: Protein deglycase DJ-1, Nrf2: Nuclear factor erythroid 2-related factor 2, SOD: Superoxide dismutase, GPx: Glutathione peroxidase, iNOS: Inducible nitric oxide synthase, NF-κB: Nuclear factor kappa-light-chain-enhancer of activated B cells, sEH: Soluble epoxide hydrolase, UPS: Ubiquitin-proteasome system, VPS35: Vacuolar protein sorting-associated protein 35, GCase: Glucocerebrosidase, ATPase 13A2: P5B-type ATPase 13A2.

PD involves multiple intertwined mechanisms that drive an imbalance between ROS and antioxidant defenses. The key affected pathways are summarized in Figure 1 and are discussed in the following sections of the text.

### 2.1. Mitochondrial Dysfunction

Pathogenic α-synuclein (α-Syn) protein, encoded by the gene *SNCA*, has been shown to localize in mitochondria, where it causes complex I disfunction and compounds ROS output [20]. Impaired mitochondrial respiration, especially in complex I, is a hallmark of PD. No matter whether it is related to α-Syn or another mechanism, it results in electron leak and then, to excess superoxide (O_2_^•^^−^) production [21].

Mitochondrial dynamics is the process of maintaining a balance between the processes of fission, fusion, and mitophagy, and is crucial for neuronal survival. Mitochondrial fission is the process of splitting mitochondria into smaller parts, regulated by the cytoplasmic GTPase Dynamin-1-like protein (Drp1). Overactive fission has been linked to decreased neuronal survival in PD, and its pharmacological inhibition is protective in experimental models [22]. It is important to distinguish between peripheral fission, which triggers mitophagy and midzone fission, supporting biogenesis. Indiscriminate Drp1 inhibition may risk impairing both maladaptive fragmentation and physiological quality-control and biogenesis. This necessitates novel therapeutic strategies that affect the fission machinery in PD be carefully pharmacologically tested [23].

Familial PD is an example that underscores the importance of *PINK1* and *PRKN* (Parkin) genes, which encode proteins that normally ubiquitinate damaged mitochondria. Then they are recognized by sequestosome-like receptors for removal. This results in the initiation of autophagosome formation and mitophagy, ultimately clearing defective mitochondria [24,25]. Defective mitophagy components lead to accumulation of damaged, ROS-generating organelles [26].

Peroxisome proliferator-activated receptor gamma coactivator 1-alpha (PGC-1α) is a protein that acts as a transcriptional coactivator, augmenting the interaction of the nuclear receptor PPAR-γ with multiple transcription factors. It is known to be the primary regulator of liver gluconeogenesis, but is also a master regulator of mitochondrial biogenesis, or the creation of new mitochondria [27]. Downregulation of PGC-1α in PD reduces the expression of respiratory enzymes and antioxidant proteins [28]. Conversely, PPAR-γ activation has been shown to promote mitochondrial biogenesis [29].

### 2.2. Redox-Active Enzymes

SN in normal brains contains lower GSH levels [30] and probably as a compensation, higher glutathione peroxidase (GPx) [31], compared to other brain regions. In PD SN neurons, GSH is further decreased and the protective function of GPx against lipid peroxidation may be depleted by redirecting it to neuromelanin production [32]. The key antioxidant enzyme NAD(P)H dehydrogenase [quinone] 1 (NQO1) detoxifies toxic quinones and can reduce ubiquinone and vitamin E quinone back to their active antioxidant hydroquinone forms [33]. It has also been shown to be upregulated in astroglial and neuronal cells of PD patients’ SN [34]. *DJ-1* is another important gene for redox balance, as it encodes a redox-sensitive chaperone that detoxifies peroxides. Moreover, under oxidative stress, DJ-1 translocates to mitochondria, where it protects against toxin-induced depolarization and helps maintain complex I activity and mitochondrial morphology [35]. Thus, the loss of DJ-1 promotes oxidative damage [36].

Peroxiredoxin-3 (PRDX3), a mitochondrial antioxidant peroxide reductase that neutralizes H_2_O_2_ and other ROS, is implicated in PD. In PD models, *LRRK2* mutations increase PRDX3 phosphorylation, reducing its activity and amplifying oxidative stress from dopamine oxidation products like dopamine quinones [37].

In contrast, the hyperfunction of enzymes that produce ROS or RNS contributes substantially to oxidative damage. Overactivated NADPH oxidases (NOX), especially NOX2 in microglia, and inducible nitric oxide synthase (iNOS) generate superoxide and nitric oxide, which combined, produce the highly deleterious peroxynitrite during neuroinflammation [38]. Elevated myeloperoxidase (MPO) in PD brain produces hypochlorous acid and other prooxidants [39].

### 2.3. Iron Metabolism and Ferroptosis

SN is iron-rich, and PD brains show abnormally high iron accumulation [40]. Excess free Fe^2+^ drives Fenton chemistry, producing highly reactive ^•^OH radicals. Dysregulated iron homeostasis due to reduced ferroportin-mediated iron export, exacerbated by ceruloplasmin (CP) oxidation and functional impairment causes the accumulation of free Fe^2+^ that catalyzes dopamine autooxidation and lipid peroxidation [41,42], potentially leading to ferroptosis, an iron-dependent cell death [43]. Clinical evidence that ferroptosis is a dominant mechanism in PD is limited and largely based on genetic-mechanistic and preclinical proof. PD neurons show features of ferroptosis and model toxins like MPTP induce ferroptosis-like death blocked by ferrostatin-1 [44]. A defective *CP* gene causes aceruloplasminemia in patients, which is a movement disorder sharing some similarities with PD [45]. Iron overload could hinder the protective GPx4 and affect PD etiology [46]. Some antiferroptotic defenses, such as the calcium-independent phospholipase A2 (iPLA_2_β), encoded by *PLA2G6* can protect membranes from iron-driven lipid peroxidation. Defective iPLA_2_β allows oxidized phospholipids to accumulate and promote ferroptosis. Mutations in *PLA2G6* can cause dystonia—parkinsonism with cognitive decline.

### 2.4. Dopamine Metabolism

Dopamine can be either protective or toxic depending on the cellular context [47]. Under physiological conditions, newly synthesized dopamine is rapidly packed into acidic vesicles, which prevents its oxidation. In PD, surviving neurons have higher dopamine turnover, producing excessive H_2_O_2_ and quinones [48]. Monoamine oxidase-B (MAO-B) on mitochondria metabolizes dopamine, releasing H_2_O_2_, which in the presence of Fe^2+^ yields the more toxic ^•^OH [49]. Enzymes like NQO1 detoxify dopamine radicals in PD [50]. When cytosolic dopamine accumulates, it is auto-oxidized to quinones such as aminochrome that drive mitochondrial dysfunction, promote toxic α-Syn protofibrils [51], and impair proteasomal and lysosomal function [52]. Aminochrome forms the polymer neuromelanin, which has antioxidant properties [53]. However, neuromelanin, which chelates metals, can become harmful when overloaded with iron. Releasing this iron from dying neurons fuels microglial activation and further increases oxidative stress [54].

### 2.5. Protein Misfolding and Aggregation

α-Syn aggregation is a major hallmark of PD. Accumulated Lewy bodies themselves contain lipid peroxidation products and oxidized proteins, evidence of in situ oxidative damage [55]. Impaired proteasomes and autophagy due to mutations and oxidative stress predispose damaged ROS-generating proteins to further accumulate [56]. ROS and RNS oxidize or nitrate α-Syn and other proteins, promoting their misfolding and oligomerization [57]. Oxidized α-Syn binds mitochondrial membranes and impairs complex I, further increasing ROS and RNS in a vicious cycle [58].

Proteostasis combines cellular processes that regulate protein synthesis, trafficking, folding, and degradation to ensure a balanced and functional proteome. It consists of molecular chaperones and cochaperones for folding and conformational maintenance, the ubiquitin-proteasome system (UPS) for targeted degradation, and the autophagy-lysosomal pathway (ALP) for clearing misfolded or aggregated proteins [59]. Within the ALP, endosomal–lysosomal trafficking machinery is crucial for delivering cargo and hydrolases to lysosomes. The retromer complex, centered on the cargo-recognition subunit VPS35, mediates endosome-to-Golgi retrieval and recycling of sorting receptors and lysosomal enzymes. *VPS35* mutations disrupt retromer assembly, cause endosomal trafficking defects, misroute proteins and impair autophagic and lysosomal degradation of α-Syn, thereby promoting its aggregation and ROS generation [60]. Small Rab GTPases act as master regulators of vesicle budding, motility, and fusion along these pathways. In PD, hyperactive LRRK2 phosphorylates a subset of Rab proteins, disturbs endolysosomal and autophagic vesicle trafficking, altering lysosomal function and further compromising α-Syn clearance [61]. Induced pluripotent stem cells (iPSC-derived PD astrocytes demonstrate that proteasome hypoactivity overall results in α-Syn accumulation [62].

### 2.6. Neuroinflammation

Chronic inflammation in PD is also both a cause and consequence of oxidative damage. Astrocytes normally supply neurons with GSH precursors, but in PD they become dysfunctional, limiting neuronal antioxidant support [63]. Decreased function of Nrf2 or its regulator DJ-1 in glia not only lowers antioxidants but also predisposes to excessive NF-κB/NLRP3 inflammation [64]. The NLRP3 inflammasome, a protein complex in microglia and other myeloid cells, controls the maturation of IL-1β and IL-18, cytokines strongly implicated in dopaminergic neurodegeneration [65]. Activated microglia release cytokines that induce oxidative enzymes in glia and neurons [66]. Microglial NADPH oxidase (NOX2) generates superoxide that can spill over to neurons [67]. MPO has also been found to be increased in PD brains [68]. Adenosine A_2A_ receptors, highly expressed on striatopallidal medium spiny neurons as well as astrocytes and microglia, enhance glutamatergic transmission and promote a pro-inflammatory microglial phenotype, leading to upregulation of NOX2 [69]. Mutant *LRRK2*, which is expressed in immune cells, drives a hyperinflammatory microglial phenotype with excess ROS production [70]. Pathogenic LRRK2 kinase activity drives microglial activation and ferroptotic cell death through dysregulation of the system Xc–GSH–GPX4 axis [71], as well as modulation of the p62–Keap1–Nrf2 signaling pathway [72].

Soluble epoxide hydrolase (sEH) is another proinflammatory enzyme related to PD etiology. It degrades anti-inflammatory epoxy fatty acids, thereby promoting inflammation and cellular stress. In PD and related α-Syn disorders, sEH levels and activity are elevated in the striatum, which is associated with more ER/oxidative stress, α-Syn phosphorylation, and dopaminergic neuron loss, while blocking or deleting sEH protects against these PD-like neurotoxic changes [73].

## 3. Genetic Contributors to Redox Imbalance in PD

PD has a complex genetic architecture [74]. Only about 5–10% of cases are due to high-penetrance mutations in single genes [75]. Well-established PD-related gene mutations include autosomal-dominant *SNCA* (previously *PARK1/PARK4*), *LRRK2* (previously *PARK8*), and *VPS35* (previously *PARK17*), and autosomal-recessive *PRKN* (previously *PARK2*), *PINK1* (previously *PARK6*), and *DJ-1* (*PARK7*) [76]. Heterozygous *GBA1* variants are also a major risk factor [77]. These genes normally support mitochondrial quality control, protein degradation, and antioxidant defenses, so their loss leads to excess ROS. For example, *DJ-1* loss impairs cellular peroxide detoxification, and defective *PINK1*/*PRKN* impairs clearance of damaged, ROS-producing mitochondria.

By contrast, most PD cases are idiopathic and polygenic. Large genome-wide association studies (GWAS) have now identified ~90 common risk loci (including in *SNCA*, *GBA1*, *LRRK2*, and *MAPT*) that collectively explain roughly 16–36% of PD heritability [78]. Table 1 demonstrates how the discussed PD mechanisms relate to some of those typical genetic mutations.

PD risk reflects a mix of rare monogenic mutations and the cumulative effect of many common variants, which alone have low predictive values [79]. Aggregating these many low-risk variants into a polygenic risk score (PRS) predicts overall disease susceptibility and correlates with age of onset: patients with higher PRS tend to develop PD at a younger age [80]. Higher PRS predicts earlier disease onset, but gene–environment interactions should also be taken into account, as they further modify risk. Therapy outcomes also vary by genotype, e.g., *PRKN* and *LRRK2* mutation carriers do particularly well with deep brain stimulation (DBS) [81], whereas *GBA1* carriers face more rapid cognitive decline post-DBS [82].

**Table 1:** Examples of important PD-associated genes related to oxidative stress and their roles in physiological and pathological circumstances.

Primary Pathway	Gene/Protein	Physiological Role	Pathological Role in PD
Synaptic vesicle trafficking & α-Syn pathology			
Synaptic vesicle cycle/Lewy pathology	***SNCA*** **(*****PARK1/4*****)—α-Syn** [83,84]	Presynaptic protein binding synaptic vesicles and curved membranes; regulates vesicle recycling; fine-tuning neurotransmitter release.	Missense mutations or gene multiplication (duplication/triplication) cause autosomal-dominant PD. Misfolded α-Syn forms oligomers/fibrils → Lewy bodies/neurites; disrupts synaptic vesicle trafficking, impairs mitochondria, overwhelms proteasome/lysosome systems. Extracellular α-Syn seeds pathology in neighboring cells and activates microglia.
Mitochondrial quality control & mitophagy			
Mitophagy (PINK1–Parkin axis)	***PINK1*** **(*****PARK6*****)** [85,86]	Ser/Thr kinase that accumulates on depolarized mitochondria; phosphorylates ubiquitin and parkin, initiating ubiquitin tagging of damaged mitochondria and recruitment of autophagy machinery (mitophagy).	Loss-of-function mutations cause autosomal-recessive early-onset PD. Impaired PINK1 signaling → defective mitophagy, accumulation of dysfunctional mitochondria, increased ROS and calcium dysregulation, heightened sensitivity to toxins (MPTP, rotenone).
Mitophagy (PINK1–Parkin axis)	***PRKN*** **(*****PARK2*****)—parkin** [87,88]	E3 ubiquitin ligase; together with PINK1, promotes proteasomal or lysosomal degradation and supports mitochondrial biogenesis (via PARIS/PGC-1α axis).	Recessive *PRKN* mutations are the most common cause of early-onset PD. Loss of parkin prevents efficient mitophagy and proteostasis → buildup of damaged mitochondria and substrates, reduced PGC-1α, defective mitochondrial biogenesis, chronic oxidative stress, and dopaminergic neuron loss.
Mitochondrial redox sensor/antioxidant	***DJ-1*** **(*****PARK7*****)** [89,90,91]	Oxidation-sensitive protein that acts as an antioxidant and redox sensor; translocates to mitochondria under stress, supports complex I activity and modulates transcription of antioxidant genes (via Nrf2, ERK).	Recessive *DJ-1* mutations → loss of antioxidant/deglycase function. Neurons become more vulnerable to oxidative stress and mitochondrial toxins; increased protein glycation, ROS, and mitochondrial dysfunction contribute to early-onset parkinsonism. Overoxidized/inactive DJ-1 is also found in sporadic PD SN.
Lysosomal cation & polyamine homeostasis	***ATP13A2*** **(*****PARK9*****)** [92,93]	P5B-type ATPase located mainly in late endosome/lysosome; exports polyamines (spermidine/spermine) from lysosomal lumen to cytosol, helping maintain lysosomal pH, providing antioxidant polyamines to buffer mitochondrial ROS	Biallelic loss-of-function causes Kufor–Rakeb syndrome (juvenile parkinsonism). Deficient ATP13A2 → polyamine trapping in lysosomes, alkalinization, impaired autophagy, increased mitochondrial ROS, and susceptibility to toxins. Iron/manganese accumulation and enhanced α-Syn aggregation are seen in models and patients, linking it to oxidative stress-driven PD.
Lysosomal—autophagy system & lipid metabolism			
Lysosomal sphingolipid metabolism	***GBA1*****—glucocerebrosidase (GCase)** [94,95]	Lysosomal β-glucocerebrosidase that hydrolyzes glucosylceramide and glucosylsphingosin glucose; maintains sphingolipid homeostasis and supports lysosomal proteostasis	Heterozygous *GBA1* mutations reduce GCase activity → accumulation of glucosylceramide/glucosylsphingosine, destabilizing lysosomal function. This promotes α-Syn misfolding/oligomerization and impairs its clearance. *GBA1* variants are the most common genetic risk factor for PD, associated with earlier onset and more rapid cognitive decline.
Endosome–lysosome trafficking/retromer	***VPS35*** **(*****PARK17*****)** [96,97,98]	Participates in synaptic trafficking. Necessary for correct trafficking of lysosomal hydrolases and receptors.	D620N and other mutations disturb trafficking of lysosomal enzymes and receptors, leading to lysosomal dysfunction, defective autophagy, and accumulation of α-Syn and damaged mitochondria.
Membrane remodeling	***PLA2G6*** **(*****PARK14*****)** [99,100]	Calcium-independent phospholipase A2; remodels phospholipids in membranes, participates in membrane repair, vesicle trafficking, and mitochondrial homeostasis.	Recessive mutations cause PARK14 parkinsonism and brain iron accumulation. Disrupted PLA2 activity leads to abnormal membrane phospholipid composition, mitochondrial swelling, axonal spheroids, and increased susceptibility to oxidative damage and α-Syn pathology.
Kinase signaling, vesicle trafficking, & neuroinflammation			
Rab kinase signaling & vesicle/lysosome regulation	***LRRK2*** **(*****PARK8*****)—dardarin** [101,102,103]	Large kinase with GTPase domains; phosphorylates Rab GTPases, regulating endolysosomal trafficking, autophagy, cytoskeleton, and innate immune responses in neurons and microglia.	Common dominant cause of late-onset PD. Pathogenic mutations (e.g., G2019S, R1441C/G) often ↑ kinase activity, causing hyper-phosphorylation of Rab proteins → mis-trafficking of endosomes/lysosomes, impaired autophagy, mitochondrial stress, and increased α-syn accumulation. In microglia, hyperactive LRRK2 promotes pro-inflammatory phenotypes and ROS/RNS production.
Metal homeostasis & oxidative stress			
Iron export/brain iron regulation	***CP*** **(ceruloplasmin)** [104,105,106]	Together with ferroportin, control iron efflux and oxidation in brain; maintain iron pools in non-toxic levels.	Variants of CP with functional impairment can contribute to regional iron accumulation (SN), enhancing Fenton chemistry and ferroptosis susceptibility in PD [107]; more clearly implicated in other brain iron accumulation dyskinetic disorders, but mechanistically relevant [45].

Arrow meaning: results in (→); inreases (↑).

## 4. Conventional Antiparkinsonian Drugs and Their Effects on Oxidative Stress

Most of the currently used PD treatments are hypothesized or known to influence redox balance via multiple mechanisms, summarized in Table 2. The adenosine A_2A_ receptor antagonist istradefylline likely mitigates neuroinflammation-mediated ROS by reducing IL-17A hypersecretion [108,109]. Dopamine receptor agonists have shown some indication of delaying the need to start levodopa treatment [110]. Aside from those two exceptions, most existing treatments either do not or marginally target the underlying oxidative stress-related pathways [111]. Drugs like levodopa with carbidopa, COMT inhibitors, and MAO-B inhibitors provide symptomatic relief and may indirectly influence redox balance, but most likely lack direct disease-modifying effects on oxidative stress-related neurodegeneration. Another partial exception is rasagiline, for which there is some clinical data from the ADAGIO trial (NCT00256204) that it might exert disease modifying effects with 1 mg/day, but not with 2 mg/day [112]. This clearly demonstrates the need for therapies that directly address the etiology of oxidative stress and neurodegeneration in PD. This observation, together with the notion of different disease genotypes, is a reason to tailor novel therapeutic approaches to patient genotypes. However, the inability of clinical trials to demonstrate disease modifying efficacy in PD could also be due to the fact that the onset of therapy follows the identification of the first symptoms, by which time unrecoverable damage to SN has likely occurred. Moreover, as demonstrated in the previous section, PD can be caused by different etiologies, converging at oxidative stress, and there may not be a single disease modifying strategy for all of them. Thus, most clinical trials have shown negative outcomes, likely due to inaccurate timing and population choice.

## 5. Research on Redox-Targeted Therapeutic Strategies in PD

Developing treatments focused on the critical pathological pathways may enable more effective management of PD progression beyond symptomatic control, provided that the appropriate patient group is specifically targeted with a relevant therapy.

Modern experimental therapies are shifting away from single antioxidant supplements, which largely failed in trials, toward strategies that restore redox balance and neuronal survival via endogenous defenses and opens the path to multi-pathway interventions (Table 3).

### 5.1. Activating Endogenous Antioxidant Pathways

A leading approach to decrease oxidative stress is to activate the Nrf2 pathway, the master regulator of cellular antioxidant responses. Dimethyl fumarate (DMF), an FDA-approved multiple sclerosis drug, exemplifies this strategy. It covalently modifies Keap1 cysteines to release Nrf2. In PD models, DMF upregulated antioxidant enzymes and attenuated α-Syn-induced dopaminergic neurodegeneration while reducing microglial inflammation [161]. Omaveloxolone (RTA-408), a synthetic oleanane triterpenoid, similarly activates Nrf2 and has shown neuroprotective effects in preclinical PD studies [162]. Other Nrf2 activators under exploration include the dietary isothiocyanate sulforaphane [163], bardoxolone (CDDO-meleate) [164], and certain curcumin derivatives [165]. Similarly, proteolysis-targeting chimeras (PROTACs) are being designed to degrade specific proteins. A peptide-based PROTAC that tags Keap1 for ubiquitination and degradation effectively stabilized Nrf2 and upregulated antioxidant genes in vitro [166]. Similarly, the expression of Nrf2 can be silenced by small inhibiting RNAs (siRNA) with potential use for preclinical modelling [167]. Nrf2 induces NQO1, which can detoxify dopamine quinones in the PD SN brain [4]. By inducing a broad spectrum of endogenous defenses, Nrf2 activators can also indirectly decrease glial inflammation, since Nrf2 activation inhibits NF-κB and cytokine production [168]. This complex enhancement of the cell’s antioxidant program is a compelling strategy, although translating it to human PD will require careful dosing to avoid side effects, e.g., DMF’s gastrointestinal and immunomodulatory effects [169].

### 5.2. Antioxidant Precursors and ROS Scavengers

Supplement antioxidants or their precursors could theoretically boost the brain’s capacity to neutralize ROS. Dietary antioxidants such as vitamins E and C [170], coenzyme Q10 (ubiquinone, CoQ10) in high doses [171], creatine [172], melatonin [173], etc., failed to demonstrate disease-modifying effects in large trials. A possible explanation could be their poor brain penetration or the fact that by the time of clinical PD, oxidative damage is too far advanced to be reversed by antioxidants. Another effective approach could be to enhance endogenous GSH. N-acetylcysteine (NAC), a GSH precursor, provides cysteine thiols to replenish intracellular GSH. In cellular and animal PD models, NAC protects dopaminergic neurons from prooxidant damage by elevating GSH. Small pilot trials in PD patients, using oral or intravenous NAC, reported increased brain GSH levels and even modest improvements in motor symptoms [174]. For example, one small study found that a course of NAC therapy improved Unified PD Rating Scale scores and increased dopamine transporter by binding on DAT scans, suggesting a possible neuroprotective effect [133]. While encouraging, these studies were not controlled; larger trials are needed, as some oral NAC studies showed only trends, likely due to bioavailability issues. Analogues of GSH or lipid-permeable GSH esters are also being explored to directly augment antioxidant reserves in neurons.

### 5.3. Mitochondria-Targeted Antioxidants

These compounds attach an antioxidant moiety like ubiquinol or vitamin E analogues to a lipophilic cation as triphenylphosphonium, which accumulates in mitochondria driven by the inner membrane potential [175]. MitoQ (mitoquinone) was one of the first such agents tested in PD. In a 12-month placebo-controled trial in early PD with around 130 patients on two doses of MitoQ vs. placebo, it was safe and well-tolerated. However, it showed no significant benefit on disease progression or symptoms compared to placebo [176]. Despite positive preclinical data, the trial indicated that simply scavenging mitochondrial ROS was insufficient to alter PD’s course, or that MitoQ’s delivery and dose were suboptimal. Other mitochondria-targeted antioxidants, such as SkQ1 [177], elamipretide (SS-31) [178], used for Barth syndrome treatment, and EPI-743 [179] are under investigation, but none have yet proven effective. These failures could suggest that antioxidants work better in prodromal stages or when targeted to key organelles and that they likely need to be combined with other therapies.

### 5.4. Iron Chelation and Ferroptosis Inhibition

Iron dysregulation in the SN is a well-documented feature of PD, contributing to ongoing oxidative injury [180]. Iron chelation therapy has thus been tested to remove iron. Deferiprone, an oral iron chelator that crosses the blood–brain barrier, was evaluated in early PD patients. A trial showed that 36 weeks of deferiprone significantly reduced nigral iron content on magnetic resonance imaging (MRI), but unexpectedly, clinical outcomes worsened. In the Phase II FAIRPARK-II study (NCT02655315), deferiprone-treated patients had worsened motor symptoms compared to placebo patients over 9 months [181]. This negative result suggests that iron in early PD may be a compensatory factor and not merely a simple driver of progression. Excessive iron removal could impede enzymes like tyrosine hydroxylase, an iron-dependent enzyme for dopamine synthesis, thereby worsening dopaminergic function [182]. This comes to show that any iron-targeted therapy must be dosed and timed carefully in order not to perturb neuronal metabolism pathologically.

Ferroptosis is a form of iron-dependent cell death caused by lipid peroxidation. Dopaminergic neurons, with their high lipid content and oxidative dynamics, may be vulnerable to this kind of cell death [46]. Ferrostatin-1, liproxstatin-1, and certain vitamin E analogues are potent lipophilic radical scavengers that can halt the associated chain reactions of lipid peroxidation [183]. In PD cell and animal models, including α-Syn overexpression and toxin models, ferroptosis inhibitors protect neurons from death by preserving membrane integrity [184]. Notably, ferroptosis also affects inflammation, as lipid peroxides can activate microglia, and inflammation can exacerbate ferroptosis as well [185]. Therefore, future trials might combine ferroptosis inhibitors with anti-inflammatory agents for synergistic protection.

### 5.5. Mitochondrial Support and Quality Control

PD is accompanied by mitochondrial dysfunction characterized by impairments in the electron transport chain, reduced ATP and increased mitochondrial ROS production [186]. CoQ10 is a metabolic supplement and a vital electron carrier in the respiratory chain. Small trials of CoQ10 showed hints of slowing decline, but a large Phase III trial (QE3) conclusively found no clinical benefit of high-dose CoQ10 in PD [171].

A recent in vivo study with a mouse paraquat model showed preserved motor and cognitive function and protected dopaminergic neurons from oxidative death after delivering the *PRDX3* (mitochondrial peroxiredoxin-3) gene, using an adeno-associated virus (AAV) vector coupled to a brain-penetrant peptide (RVG9R) [134].

Activation of the AMPK–PGC-1α–SIRT1 pathway in PD models improves mitochondrial function and antioxidant capacity [187]. NAD^+^ is essential for mitochondrial enzymes and for activating sirtuins (SIRT1) and the PGC-1α pathway. SIRT1 is a NAD^+^ -dependent deacetylase, which reacts to changes in the cell’s energy status [188]. Together, they induce mitochondrial biogenesis and antioxidant gene expression.

NAD^+^ boosters have gained significant interest after the randomized NADPARK trial, which showed that 1000 mg of nicotinamide riboside (NR) daily for 30 days in early PD patients was well tolerated and raised cerebral NAD^+^ levels [16]. Notably, there were trends towards mild clinical improvement. Gene expression analyses indicated that NR upregulated mitochondrial and lysosomal pathways in peripheral tissues. NR-treated patients also showed altered brain glucose metabolism on PET, suggesting improved or normalized neuronal metabolic activity. Moreover, they had reduced inflammatory cytokines in the cerebrospinal fluid [189]. These findings demonstrate that NAD^+^ repletion is a potentially effective disease-modifying approach. NAD^+^ precursors as nicotinamide mononucleotide (NMN) and SIRT1 activators as resveratrol, urolithin A [190], or the more potent experimental drug SRT2104 are being researched as energy-redox regulators [191]. This approach could mimic some benefits of regular aerobic exercise [192] and caloric restriction [193], known to activate AMPK–PGC-1α–SIRT1 and to improve mitochondrial function, antioxidant capacity, and mitochondrial dynamics in PD models [194].

Mitochondrial dynamics is another target for PD treatment. Mitochondrial Division Inhibitor-1 (Mdivi-1) is a small molecule that inhibits the fission regulator Drp1, promoting a more fused mitochondrial network [195]. Remarkably, it has shown neuroprotective effects in a rat PD model, overexpressing mutant A53T-α-Syn [22]. Chronic Mdivi-1 treatment preserved striatal dopamine levels and completely prevented the progressive motor deficits seen in untreated PD rats [196]. Post-mortem analysis confirmed that Mdivi-1–treated rats had significantly more nigral dopaminergic neurons and fibers remaining, indicating protection of the nigrostriatal pathway. The compound also reduced markers of oxidative stress. These results highlight that normalizing mitochondrial morphology and preventing excessive fission can attenuate neurodegeneration [197]. Mdivi-1 itself is not yet in human trials and questions remain about its specificity towards peripheral and midzone fission. Still, it provides proof-of-concept that targeting mitochondrial dynamics is a viable neuroprotective strategy.

Patients with some *PINK1* and *PRKN* mutations have impaired mitophagy, leading to accumulation of ROS-producing mitochondria [198]. Parkin agonists and PINK1 small molecule activators aim to boost this clearance pathway. Key compounds include MTK458 and kinetin riboside, which enhance mitophagy, protect neurons, and show potential in preclinical PD models [199]. Another approach is inhibiting negative regulators of mitophagy, such as the deubiquitinase USP30. In cellular models, USP30 inhibition stabilizes Parkin’s ubiquitin tags on dysfunctional mitochondria, thereby promoting their autophagosome engulfment [200]. For example, in human midbrain neurons derived from iPSCs, including neurons lacking functional Parkin, a selective USP30 inhibitor restored mitophagy and significantly reduced oxidative stress levels and neuronal viability [201].

### 5.6. Enhancing Proteostasis and Lysosomal Function

Oxidatively damaged proteins and organelles can themselves promote more ROS generation, so removing them is an attractive therapeutic modality in PD treatment. Proteostasis enhancers include a variety of agents as small-molecule autophagy inducers. Such are trehalose, metformin, or mTOR inhibitors that promote removal of protein aggregates and defective mitochondria [202]. Proteasome activators or deubiquitinase inhibitors beyond USP30 also facilitate the degradation of misfolded proteins [203]. By clearing toxic waste from cells, these treatments aim to indirectly reduce oxidative stress and inflammation [204]. Ambroxol, a mucolytic drug, has been found to act as a molecular chaperone for glucocerebrosidase (GCase) [205,206]. A recent phase II trial (AiM-PD) showed ambroxol was safe, penetrated the CNS, and increased cerebrospinal fluid (CSF) GCase levels of PD patients [207,208]. In parallel, gene therapy approaches like AAV-based *GBA1* gene therapy (PR001 or LY3884961) are being clinically tested to augment GCase directly in the brain [209,210]. These *GBA*-targeted therapies could represent tailored interventions for a subgroup of PD with prominent lysosomal deficits.

### 5.7. Targeting Neuroinflammation and Redox Crosstalk

Neuroinflammation and oxidative stress in PD form a vicious cycle, so targeting inflammatory pathways can have redox benefits, too. Broad anti-inflammatories as NSAIDs did not alter PD progression [211], so current research is focusing on more specific pharmacological modulation.

In PD models, blocking the NLRP3 inflammasome reduces microglial activation and neuronal loss [212]. A recent study of dl-3-n-butylphthalide (NBP), a multi-target drug originally used in stroke, showed that NBP can inhibit NLRP3 inflammasome activation in PD models [154]. In MPTP mice and 6-OHDA cell models, NBP treatment suppressed NLRP3 activation, reduced IL-1β/IL-18 release, and ameliorated mitochondrial impairments, leading to reduced ROS, rescue of dopaminergic neurons, and improved motor function [154]. NBP is now in clinical testing in China for PD, although its multi-faceted effects make it hard to pinpoint how much of the benefit comes from NLRP3 inhibition versus other actions [155]. Another experimental drug with such effects is the small molecule inhibitor MCC950, which is effective in a α-Syn mouse PD model [213].

The soluble epoxide hydrolase (sEH) enzyme is also a pharmacological target, as it links epoxyeicosatrienoic acids (EETs), metabolism, and inflammation. By preserving anti-inflammatory EETs signaling, sEH inhibitors might indirectly protect neurons from oxidative damage [73]. In models of PD, sEH levels were found to be elevated, especially in cases with *PRKN* mutations [73]. Treatment of *PRKN*-mutant dopaminergic neurons with the sEH inhibitor TPPU was shown to prevent oxidative stress-induced apoptosis [73]. A recent study in a MPTP mouse PD model also suggested that genetic deletion or inhibition of sEH reduced dopaminergic neurodegeneration [214]. Currently, the sEH inhibitor EC5026 is in early clinical trials [215].

*LRRK2* is highly expressed in microglia and peripheral macrophages; its overactivity alters vesicle trafficking via Rab GTPases, promoting a pro-inflammatory phenotype and impairing ALP protein clearance [149]. LRRK2 kinase inhibitors, such as BIIB122/DNL151, DNL201, represent another targeted approach at the intersection of inflammation and redox dysfunction. Mutations in *LRRK2* cause familial PD, but even in sporadic PD, LRRK2 kinase activity is often elevated. By inhibiting LRRK2, these drugs aim to reduce microglial inflammatory output and improve neuronal proteostasis. Several LRRK2 inhibitors are in phase I–II trials [216]. They have shown good safety so far, and one of them, DNL151, is progressing to a larger trial to test if it can slow clinical progression in PD [217].

Another avenue is NF-κB pathway inhibition. NF-κB is a master transcription factor for many pro-inflammatory mediators. Some Nrf2 activators, like DMF, concurrently inhibit NF-κB, and compounds such as salsalate, curcumin, or berberine have mild NF-κB inhibiting properties that might be beneficial, though evidence in PD patients is limited [218].

GLP-1 receptor agonists as exenatide and liraglutide are incretin mimetics. It has emerged from epidemiological observations that there might be reduced PD incidence in diabetic patients on GLP-1 drugs [219]. These peptides, originally used for treating diabetes, cross the blood–brain barrier, have anti-inflammatory effects, and improve mitochondrial function. They enhance insulin/IGF-1 signaling in the brain, which can improve neuronal glucose utilization and may upregulate neurotrophic factors [220]. They have been shown to reduce microglial activation [221]. A Phase II trial of weekly exenatide in moderate PD showed that patients on exenatide maintained better motor scores compared to placebo, even 12 months after stopping the drug, suggesting a possible disease-modifying effect [158]. However, the definitive Phase III trial failed to show any slowing of PD progression with exenatide over 2 years [159]. The treated and placebo groups’ motor progression was virtually identical, indicating that exenatide does not meaningfully alter the neurodegenerative trajectory. However, those findings could benefit from being reevaluated after careful patient stratification.

There is growing interest in “senolytic” therapies for PD. Cellular senescence in glia and some neurons can lead to a pro-inflammatory, pro-oxidant secretory phenotype because senescent cells secrete the so-called Senescence-Associated Secretory Phenotype (SASP) factors, driving chronic inflammation and oxidative stress [222]. Clearing these senescent cells with senolytic drugs, like the combination of dasatinib and quercetin, has shown efficacy in other age-related diseases [223]. It might reduce chronic inflammation and oxidative stress in the aging PD brain. However, there is no data that clearly demonstrates this in PD models.

## 6. Personalized Approaches to Innovative PD Treatments

PD is a set of overlapping molecular subtypes rather than a single disorder. Different genotypes give rise to recognizable clinical phenotypes, for example, early-onset “pure nigral” *PINK1*/*PRKN* disease versus malignant, dementia-prone *SNCA* triplication or *GBA1*-PD, making them candidates for tailored pharmacological and lifestyle interventions [224].

Lysosomal-targeted strategies are mechanistically best matched to *GBA1*-linked PD and related lysosomal subtypes. *GBA1*-linked PD typically draws onset earlier and induces a faster cognitive decline. It is likely exacerbated in *VPS35* D620N or high-α-Syn phenotypes, e.g., *SNCA* multiplications. Ambroxol could be a promising approach in those patients, although a recent small clinical trial demonstrates little clinical benefit so far [208]. AAV9-based *GBA1* gene therapy is being clinically tested in *GBA1*-mutation carriers, but results are yet to be published [225]. In cases where lysosomal function is exacerbated by LRRK2 hyperactivity, its pharmacological inhibition could be an effective modality in combined therapy.

Mitochondria- and mitophagy-focused interventions align most naturally with *PINK1*- and *PRKN*-associated PD, and to some extent with *DJ-1* mutations. Clinically, *PINK1*/*PRKN* PD usually presents in early adulthood with predominantly motor symptoms, slow progression, preserved cognition, and good levodopa responsiveness, pointing to a relatively “pure” nigral mitochondrial cytopathy [226]. NAD^+^ precursors, together with SIRT1/AMPK activators and modulators of mitochondrial dynamics (e.g., Drp1 inhibitors), are conceptually best suited to such genotypes and may secondarily benefit genotypes with predominantly lysosomal dysfunction, as they result in α-Syn and in secondary mitochondrial stress and ROS production. However, beyond early biomarker trials, disease-modifying efficacy remains to be demonstrated.

Iron-driven lipid peroxidation and ferroptosis provide another mechanism, relevant in iron-overload-related phenotypes. Ferroptosis inhibitors are neuroprotective in PD models [227]. The iron chelator deferiprone reduces nigrostriatal iron but causes minor motor worsening. This could suggest that iron depletion should be dosed cautiously and only in patient groups with a positive benefit-risk balance, i.e., in later disease stages, with some gene variants of *CP*, causing systemic iron overload syndromes or genotypes with pronounced α-Synucleinopathy and *SNCA* triplications. However, direct evidence supporting such an approach is still missing.

Neuroinflammation-predominant phenotypes are mechanistically linked to *LRRK2* and *GBA1* variants, where immune cells and glia are heavily involved. *LRRK2* is highly expressed in microglia, and pharmacological *LRRK2* inhibition reduces pro-inflammatory cytokines, ferroptosis markers, and dopaminergic neuron loss in MPTP and α-Syn models [71]. GLP-1 receptor agonists showed encouraging neuroprotective and anti-inflammatory signals in early PD trials, but the large phase III Exenatide–PD3 study in an unstratified PD cohort did not slow disease progression versus placebo, indicating that any benefit may be confined to subgroups with high inflammatory load and insulin-resistant PD [228]. NLRP3 inflammasome, sEH inhibitors, and senolytic drugs targeting senescent microglia and astrocytes could be the most beneficial inflammatory phenotypes, but data about their efficacy in PD is still scarce.

**Figure 2:**
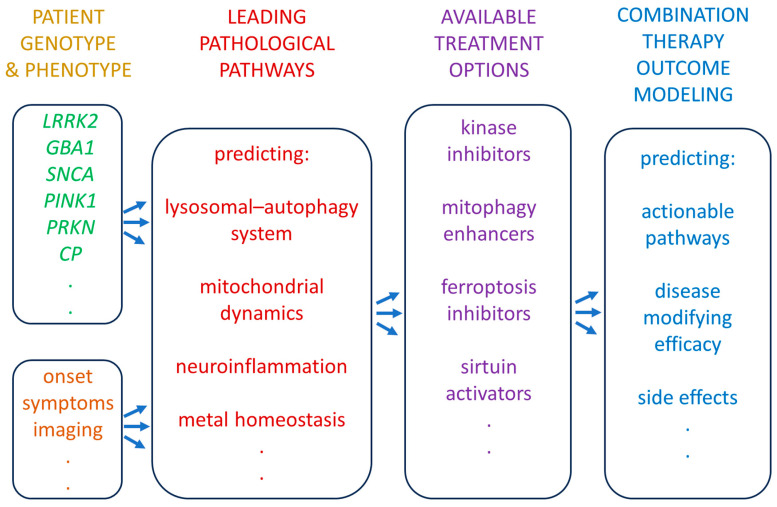
Conceptual framework for a future personalized approach, integrating information about patient phenotype and genotype to predict the leading pathological pathways, and based on data on the efficacy of available treatment options to model therapeutic outcomes. The included list of elements is not exhaustive and serves to exemplify the workflow. Abbreviations: *LRRK2*: Leucine-rich repeat kinase 2, *GBA1*: Glucosylceramidase beta 1, *SNCA*: Synuclein alpha, *PINK1*: PTEN-induced kinase 1, *PRKN*: Parkin rein-type E3 ubiquitin protein ligase (also known simply as parkin protein 2), *CP*: ceruloplasmin.

The presence of PD subtypes with different therapeutic response has been clearly demonstrated with nonpharmacological treatments like DBS. Patients with *PINK1*- and *PRKN*-associated early-onset PD, with predominantly motor features and preserved cognition, as well as those with *LRRK2* G2019S mutation, generally exhibit good to excellent motor outcomes [229]. By contrast, *GBA1* mutation carriers, who already have a higher baseline risk of cognitive decline, show an increased likelihood of faster cognitive deterioration or psychosis after DBS, although there is uncertainty whether this could be attributed to the natural course of the disease in this subgroup [230]. Reliance on peripheral oxidative stress readouts alone is unlikely to resolve brain subtype heterogeneity, so stratification efforts should prioritize CNS-proximal biomarkers or multimodal panels that explicitly bridge peripheral redox tone to central neurodegenerative processes [231]. With the accumulation of clinical and mechanistic data, the outcome of such an approach could be expected to provide useful input for modeling systems with high translation potential, conceptually outlined in Figure 2.

There are high expectations that multi-omic and machine learning approaches are beginning to operationalize the mechanistic stratification into predictive tools with potential pharmacotherapeutic applications. Patients with PD show wide interest in extensive genetic testing, which could help incorporate genome sequencing into routine clinical care [232]. Large longitudinal cohorts such as the Parkinson’s Progression Markers Initiative (PPMI) collect genomic, transcriptomic, proteomic, metabolomic, imaging, and digital clinical data, and are being used to train models that classify subtypes and forecast progression [233]. Studies combining PRS with imaging markers (e.g., dopamine transporter scans, structural MRI) and clinical features improve statistical prediction of PD risk, age at onset, and short-term progression in comparison with clinical data alone. However, individual-level prognostic certainty is still modest [234]. In the longer term, integrating a patient’s monogenic status, PRS percentile, CSF, and imaging biomarkers, as well as digital phenotype, into such models could enable “virtual testing” of treatment combinations and lifestyle interventions for the specific molecular cascades that are predominantly driving the specific individual’s disease.

## 7. Conclusions

Oxidative stress is a central downstream driver of dopaminergic neuron loss in the SN, representing a common endpoint where diverse pathogenic influences converge. Genetic mutations and environmental factors can disrupt mitochondrial function, promote neuroinflammation, impair lysosomal and autophagic clearance, and alter protein and lipid turnover, leading to the accumulation of dysfunctional cellular components. Together, these disturbances amplify ROS generation and oxidative damage, positioning ROS as a key integrator of multiple pathological processes in PD. Since PD is biologically heterogeneous and patients differ in the predominance of mitochondrial, inflammatory, lysosomal, or proteostatic dysfunction, future therapies must move beyond a “one-size-fits-all” approach. Effective disease modification will likely require early, mechanism-based interventions that bolster endogenous antioxidant and stress-response systems, complemented by rational combination strategies that target several nodes of pathology at once. Importantly, the current clinical and translational pipeline suggests that PD is entering a phase in which multiple potentially disease-modifying strategies are becoming available, targeting mitochondrial bioenergetics and quality control, lysosomal function, iron handling, ferroptosis, and inflammatory–redox crosstalk. This shift will be driven not only by new compounds, but by improved trial design: selecting the right patients, confirming CNS target engagement, and intervening earlier in the disease course. Aligning treatments with individual mechanistic profiles, using biomarkers and systems-level analyses, offers a path toward interventions that not only alleviate symptoms but also slow or alter the course of neurodegeneration.

## Figures and Tables

**Table 2:** Effects of conventional therapeutic drugs for PD on oxidative stress mechanisms. Abbreviations: PD, Parkinson’s disease; ROS, reactive oxygen species; DA, dopamine; COMT, catechol-O-methyltransferase; MAO-B, monoamine oxidase B; H_2_O_2_, hydrogen peroxide; NO, nitric oxide; NCT, ClinicalTrials.gov identifier; ISRCTN, International Standard Randomized Controled Trial Number.

Therapeutic Class	Representative Drug(s)	Effects on Redox State and Clinical Dimensions
Dopamine precursor + peripheral decarboxylase inhibitor	Levodopa/Carbidopa	Central dopamine formed from levodopa can increase oxidative stress locally, while improved motor activity may indirectly support mitochondrial function; overall considered largely redox-neutral to mildly pro-oxidant at nigral level [113]. It is the gold-standard symptomatic treatment; Long-term use leads to motor fluctuations and dyskinesias. **Clinical Trial, ELLDOPA (NCT00004733); LEAP (ISRCTN30518857); no disease modifying efficacy, increased peripheral ROS markers** [114]
Non-ergot dopamine agonists	Pramipexole	Preclinical data suggest mitochondrial protective and ROS-lowering effects by scavenging of free radicals and reduced mitochondrial permeability transition. It is often considered potentially antioxidant/neuroprotective and inhibits ferroptosis in models [115]. Improves motor symptoms and reduces “off” time; useful as monotherapy early or adjunct later; may improve depressive symptoms but increases risk of impulse-control disorders, hallucinations, and somnolence. **Clinical trial, Phase IV, PROUD (NCT00321854), no disease modifying efficacy proof** [116,117]
	Ropinirole	Similar to pramipexole with some evidence of reduced oxidative stress and mitochondrial protection in experimental models, but clinically used purely as symptomatic agent. **A clinical Trial shows no disease modifying efficacy proof** [118]
COMT inhibitors (levodopa extenders)	Entacapone	By inhibiting peripheral levodopa breakdown, may reduce peripheral oxidative metabolites; central redox impact is indirect, by more stable levodopa levels, but also more DA in brain. Used as adjunct to levodopa to reduce wearing-off and increase “on” time; little intrinsic effect on non-motor symptoms; can worsen dyskinesia due to higher effective levodopa exposure [119]. **Clinical trial, Phase III, STRIDE-PD (NCT00099268), add-on to carbidopa/levodopa, no disease modifying efficacy proof**
	Tolcapone	Similar to entacapone, but both peripherally and centrally acting. However, experimental antioxidant/mitochondrial-protective effects are offset by hepatic toxicity risk [120].
	Opicapone	Long-acting peripheral COMT inhibition with similar redox profile to entacapone [121]. **Clinical trial, Phase III, EPSILON study (NCT04978597), no disease modifying efficacy proof**
Irreversible MAO-B inhibitors	Selegiline	By blocking MAO-B, reduces oxidative deamination of dopamine and thereby lowers H_2_O_2_ generation from DA metabolism; potential antioxidant effect in nigrostriatal system, though amphetamine-like metabolites may introduce other stresses [122]. Causes mild symptomatic benefit, especially in early PD and reduction of off-time as adjunct to levodopa. Minimal direct impact on cognition; may worsen insomnia, jitteriness. There are some reports of modest delay in need for levodopa [123]. **Clinical Trial, DATATOP 1987 study, selegiline, no disease modifying efficacy proof** [124]; **a later study demonstrated proof of delayed need for levodopa** [123]
	Rasagiline	Similar to selegiline, with additional mitochondrial-stabilizing effects reported in models, often considered redox-protective, antiapoptotic and neuroprotective experimentally [125]. Generally well tolerated. Early trials hinted at possible disease-modifying effects, but this remains controversial [126]. **ADAGIO trial (NCT00256204), potential disease modifying effect with 1 mg/day, but not with 2 mg/day. Patient stratification likely required** [112]
Reversible MAO-B inhibitor with antiglutamatergic action	Safinamide	MAO-B inhibition lowers DA-derived H_2_O_2_ [127]; additional modulation of excessive glutamate release can reduce excitotoxicity and downstream oxidative damage, which is overall favorable for redox balance in models [128]. **Clinical Trial, Phase IV (NCT03944785), no disease modifying efficacy proof;**
Adenosine A_2A_ receptor antagonist	Istradefylline	Causes A_2A_ blockade on striatopallidal neurons and glia; can dampen microglial activation and neuroinflammatory signaling, which is indirectly antioxidative, resulting in less NO/ROS from activated glia, though human redox data are limited [108]. It is used as adjunct to levodopa. Reduces daily off-time and improves “off” motor function. **Clinical trial, Phase II (NCT00199433), no disease modifying efficacy proof** [129].

Clinical proof presented in bold script.

**Table 3:** Novel drugs under research for disease-modifying effects in PD treatment by the mechanism of affecting oxidative stress mechanisms. Abbreviations: AMPK, AMP-activated protein kinase; AAV, adeno-associated virus; BBB, blood–brain barrier; CNS, central nervous system; CoQ10, coenzyme Q10; DMF, dimethyl fumarate; Drp1, dynamin-related protein 1; ETC, electron transport chain; Fe^2+^, ferrous iron; GLP-1, glucagon-like peptide-1; GSH, glutathione; HO-1, heme oxygenase-1; IGF-1, insulin-like growth factor 1; IL, interleukin; ISRCTN, International Standard Randomized Controled Trial Number; Keap1, Kelch-like ECH-associated protein 1; LRRK2, leucine-rich repeat kinase 2; NAC, N-acetylcysteine; NAD^+^, nicotinamide adenine dinucleotide; NCT, ClinicalTrials.gov identifier; NF-κB, nuclear factor kappa B; NLRP3, NLR family pyrin domain-containing 3; NMN, nicotinamide mononucleotide; NQO1, NAD(P)H:quinone oxidoreductase 1; NR, nicotinamide riboside; Nrf2, nuclear factor erythroid 2–related factor 2; PROTAC, proteolysis-targeting chimera; PRDX3, peroxiredoxin-3; ROS, reactive oxygen species; sEH, soluble epoxide hydrolase; siRNA, small interfering RNA; SIRT1, sirtuin 1; SOD, superoxide dismutase.

Pharmacological Group	Representative Substance(s)	Pharmacological Mechanisms and Effects on Redox State
Endogenous redox balance & iron homeostasis		
Nrf2 pathway activators	Dimethyl fumarate (DMF), omaveloxolone, sulforaphane, bardoxolone, curcumin derivatives, Keap1-targeting peptides, Keap1-degrading PROTACs, Keap1 siRNA	Molecules inactivated by different mechanisms: blocking Keap1 → activates Nrf2 → ↑ expression of broad antioxidant and cytoprotective endogenous molecules: GSH synthesis enzymes, HO-1, SOD, catalase, NQO1 [130]. NQO1 also detoxifies dopamine-derived quinones formed by dopamine auto-oxidation [131]. Overall, this transcription shift also reduces glial inflammation [132]. **Clinical Trial, Phase II (NCT05084365) sulphoraphane, no safety or efficacy data yet; Phase II (NCT01383161) submicron dispersed curcumin, improvements in memory and attention;**
GSH precursors and analogs	N-acetylcysteine (NAC), GSH esters/analogs	NAC supplies cysteine/thiol groups → ↑ intracellular and brain GSH; protects dopaminergic neurons in models; small PD trials show ↑ brain GSH and modest motor benefit; GSH analogs similarly aim to restore GSH and neutralize ROS [133]. **Clinical Trial, Phase II (NCT02445651) NAC, 13% improved nonmotor and motor score; Phase II (NCT02655315) NAC, mild to moderate tremor worsening; raises brain GSH** [133]
Iron chelators	Deferiprone	Oral BBB-penetrant Fe^2+^ chelator → lowers nigral iron and Fenton chemistry-driven hydroxyl radical formation. **Clinical Trial, Phase IIa, FAIR-PARK-I (NCT00943748) deferiprone, reduction in brain iron and motor symptoms; Phase IIb, FAIRPARK II (NCT02655315) deferiprone, motor symptom worsening; failure likely due to inappropriate patient stratification by phenotype/genotype and disease stage.**
Ferroptosis inhibitors/lipophilic radical scavengers	Ferrostatin-1, liproxstatin-1, vitamin E analogs	Trap lipid radicals and block iron-dependent lipid peroxidation (ferroptosis); protect dopaminergic neurons from lipid ROS in PD models [107]; promising for limiting membrane oxidative damage (preclinical). **Clinical Trial, DATATOP 1987 study, tocopherol, no clinical benefit** [124].
Antioxidant gene therapy	*PRDX3* gene therapy (AAV8/RVG9R-PRDX3)	Viral delivery of mitochondrial peroxiredoxin-3 → boosts enzymatic breakdown of H_2_O_2_ and peroxides; in PD mouse model improved motor/cognitive performance and protected SN neurons from oxidative death. **Preclinical proof** [134]
Mitochondrial function & dynamics		
Mitochondria-targeted antioxidants	MitoQ, SkQ1, SS-31 (elamipretide), EPI-743	Ubiquinone/other antioxidants linked to lipophilic cations accumulate in mitochondria and scavenge mitochondrial ROS [135]. **Clinical Trial, Phase II, NCT00329056, MitoQ (Mitoquinone), marginally slowing disease progression, but not statistically significant; Phase IIa, NCT01923584, EPI-743 (Vatiquinone), decreased CNS oxidative stress biomarkers and improved motor function approaching statistical significance** [136]
NAD^+^ boosters	Nicotinamide riboside (NR), nicotinamide mononucleotide (NMN)	Raise cellular and brain NAD^+^ levels → activate SIRT1/PGC-1α, improve mitochondrial bioenergetics and antioxidant gene expression [137]. **NADPARK trial: NADPARK (NCT03816020), NR, mild clinical improvement; Phase III, NOPARK (NCT03568968), NR, no efficacy data yet**
SIRTUIN/AMPK—PGC-1A activators	Resveratrol, SRT2104, Urolithin A, bezafibrate, exercise	Activate SIRT1 and/or AMPK–PGC-1α axis → ↑ mitochondrial biogenesis, respiration and antioxidant enzymes; rebalance mitochondrial redox tone and enhance resilience to oxidative stress [138]. **Clinical Trial, Phase III, SPARX3, (NCT04284436) Exercise, potential slowing of neurodegeneration** [139]
CoQ10 (ubiquinone) supplements	Coenzyme Q10	Supports ETC and reduce electron leak/ROS [140]. **Clinical Trial, Phase II, (NCT00004731) CoQ10, potential slowing of neurodegeneration; Clinical Trial, Phase III, (NCT00740714) CoQ10, no clinical benefit, fails to replicate Phase II trial.**
Modulators of mitochondrial dynamics	Mdivi-1	Inhibits Drp1-mediated mitochondrial fission → less fragmentation, preserved mitochondrial integrity, ↓ ROS; in α-syn PD rats, restored striatal dopamine and motor function, highlighting benefit of normalizing mitochondrial morphology. **Preclinical proof** [141,142].
Mitophagy enhancers	USP30 inhibitors: USP30i-37; MTX652, parkin activators: FB231; PINK1 activators: MTK458	Parkin/PINK1 activators aim to restore defective mitophagy and remove ROS-producing mitochondria [143]. USP30 inhibition promotes parkin-mediated mitophagy by preventing de-ubiquitination of damaged mitochondria; in human (incl. *PRKN*-/-) neurons ↑ mito clearance and markedly ↓ ROS [144]. **Clinical Trial, Phase I, (NCT06579300) ABBV-1088, no safety or efficacy data yet; Phase I, (ISRCTN20898392), MTX325, no safety or efficacy data yet.**
Proteasomal function & lysosomal trafficking		
Proteostasis enhancers	Autophagy inducers: Trehalose; Memantine; Metformin; UPS modulators: MTX325-101, E3-recruiting chimeras (PROTACs)	Boost clearance of misfolded/aggregated proteins and damaged organelles via ALP and UPS systems; by removing oxidatively damaged components, indirectly reduce cellular ROS sources and redox stress [145]. **Clinical Trial, Phase I, (ISRCTN20898392) MTX325, no safety data yet; Phase III, NCT07055958, metformin as add-on to levodopa, no safety or efficacy data yet; Phase II/III, NCT07229651, metformin as add-on to Levodopa/Carbidopa; Phase III, COBALT (ISRCTN79794378), memantine as add-on to cholinesterase inhibitor; Phase II, NCT05355064, trehalose, no safety or efficacy data yet.**
Lysosomal enhancers/GBA-targeted therapies	Ambroxol, PR001A (*GBA1* gene therapy)	Ambroxol ↑ glucocerebrosidase activity, improving lysosomal degradation of α-syn and damaged organelles [146]; enhanced lysosomal flux reduces accumulation of ROS-producing substrates and alleviates secondary oxidative stress [147]. **Clinical Trial, Phase IIA, AiM-PD (NCT02941822) ambroxol, modest improvement; Phase II (NCT02914366), ambroxol, genetically stratified, stabilizes PD dementia; Phase III, ASPro-PD (NCT05778617), ambroxol, (genetically stratified), no safety or efficacy data yet; Phase I/IIa, PROPEL (NCT04127578), LY3884961 (PR001A) (genetically stratified), no safety or efficacy data yet.**
Neuroinflammation		
Kinase inhibitors	LRRK2 inhibitors: BIIB122 (also known as DNL151), DNL201, NEU-411;	*LRRK2* is highly expressed in microglia and peripheral myeloid cells, and elevated LRRK2 kinase activity increases phospho-Rab levels in these cells [148]. Altered Rab signaling also impairs endolysosomal and autophagy pathways (Rab8/10/7L1 axis), leading to defective lysosomal clearance of proteins and damaged organelles and contributing to α-syn accumulation and dopaminergic neuron vulnerability [149,150]. **Clinical Trials, Phase IIb, LUMA (NCT05348785), BIIB122 (DNL151)** [151]; **Phase II, NEULARK (NCT06680830), NEU-411** [152] **(genetically stratified), no efficacy data yet; Phase I, (2024-516888-84-00), ARV-102, no efficacy data yet.**
Suppressors of pro-oxidant cytokines production	NLRP3 inflammasome inhibitors: NT-0796 (NodThera), Dapansutrile (OLT1177), MCC950; NF-κB inhibitors: Dimethyl fumarate, salsalate, curcumin, resveratrol	Suppress formation of pro-oxidant cytokines (IL-1β, IL-18, etc.) and microglial activation; indirectly lower ROS production from activated immune cells [153]. **Clinical Trial, Phase Ib, NT-0796 (EU CT # 2023-503203-29-00) decreased neuroinflammation markers, no efficacy data yet** [153].
Pleiotropic antioxidant—anti-inflammatory drug	dl-3-n-butylphthalide (NBP)	Inhibits NLRP3 inflammasome, reduces IL-1β/IL-18 and oxidative stress, and ameliorates mitochondrial impairment in PD models; results in dopaminergic neuron rescue and behavioral improvement [154]. **Clinical Trial (ChiCTR1800018892)** [155], **motor improvements, inconclusive.**
GLP-1 receptor agonists (from inflammatory angle)	Exenatide, liraglutide	They ross BBB; improve brain insulin/IGF-1 signaling, enhance mitochondrial function and reduce neuroinflammation; overall act as modulators of mitochondrial metabolism and redox-inflammatory balance [156]. Also reduce microglial activation and pro-inflammatory signaling in brain [157]. Small trials. **Clinical Trial, Phase II,** **EXENATIDE-PD** (**NCT01971242), sustained motor benefit off-drug** [158]. **Phase III, Exenatide-PD3 (NCT04232969)** [159], **no benefits, failure probably due to lack of patient stratification.**
Soluble epoxide hydrolase (sEH) inhibitors	TPPU	Block sEH, thus shifting lipid mediators away from pro-inflammatory diols; protect iPSC-derived dopaminergic neurons (incl. *PRKN* mutants) from oxidative apoptosis [73]; link lipid inflammatory signaling to oxidative injury. **Preclinical proof** [73].
Senolytics and anti-senescence strategies	Experimental senolytics: Dasatinib + Quercetin	Clear senescent glial/neuronal cells that chronically release pro-inflammatory and pro-oxidant factors; expected to reduce background neuroinflammation and oxidative stress burden in PD. **Hypothetical rationale** [160].

Clinical/non-clinical proof presented in bold script. Arrow meaning: results in (→); inreases (↑); decreases (↓).

## Data Availability

No new data were created or analyzed in this study. Data sharing is not applicable to this article.

## Abbreviations

The following abbreviations are used in this manuscript:

PD	Parkinson’s disease
SN	Substantia nigra
ROS	Reactive oxygen species
RNS	Reactive nitrogen species
ATP	Adenosine triphosphate
GSH	Glutathione
CNS	Central nervous system
α-Syn	Alpha-synuclein
*SNCA*	Alpha-synuclein gene (synuclein alpha)
GPx	Glutathione peroxidase
GPX4	Glutathione peroxidase 4
NQO1	NAD(P)H dehydrogenase [quinone] 1
DBS	deep brain stimulation
*DJ-1*	Protein deglycase DJ-1
PRDX3	Peroxiredoxin-3
NOX	NADPH oxidase
NOX2	NADPH oxidase 2
iNOS	Inducible nitric oxide synthase
MPO	Myeloperoxidase
Fe^2+^	Ferrous iron (Fe^2+^)
^•^OH	Hydroxyl radical
MPTP	1-Methyl-4-phenyl-1,2,3,6-tetrahydropyridine
MPP^+^	1-Methyl-4-phenylpyridinium
TH	Tyrosine hydroxylase
AADC	Aromatic L-amino acid decarboxylase
VMAT2	Vesicular monoamine transporter 2
MAO-B	Monoamine oxidase B
ETC	Electron transport chain
UPS	Ubiquitin-proteasome system
ALP	Autophagy-lysosomal pathway
*VPS35*	Vacuolar protein sorting-associated protein 35
Rab	Ras-related in brain (Rab small GTPase family)
*LRRK2*	Leucine-rich repeat kinase 2
iPSC	Induced pluripotent stem cell
Nrf2	Nuclear factor erythroid 2-related factor 2
Keap1	Kelch-like ECH-associated protein 1
NF-κB	Nuclear factor kappa-light-chain-enhancer of activated B cells
NLRP3	NOD-, LRR- and pyrin domain-containing protein 3
IL-1β	Interleukin-1 beta
IL-18	Interleukin-18
iPLA_2_β	Group VI calcium-independent phospholipase A2
A_2A_	Adenosine A_2A_ receptor
sEH	Soluble epoxide hydrolase
ER	Endoplasmic reticulum
*PINK1*	PTEN-induced kinase 1
Parkin	Parkin E3 ubiquitin ligase (protein)
*PRKN*	Parkin RBR E3 ubiquitin-protein ligase (parkin gene)
GBA	Glucocerebrosidase gene
*GBA1*	Glucocerebrosidase beta 1 gene
GCase	Glucocerebrosidase (enzyme)
*ATP13A2*	P5B-type ATPase 13A2
*PLA2G6*	Group VI calcium-independent phospholipase A2 gene
*CP*	Ceruloplasmin
PGC-1α	Peroxisome proliferator-activated receptor-γ coactivator-1 alpha
PPAR-γ	Peroxisome proliferator-activated receptor gamma
AMPK	AMP-activated protein kinase
SIRT1	Sirtuin 1
NAD^+^	Nicotinamide adenine dinucleotide (oxidized form)
NR	Nicotinamide riboside
NMN	Nicotinamide mononucleotide
HO-1	Heme oxygenase-1
SOD	Superoxide dismutase
CoQ10	Coenzyme Q10 (ubiquinone-10)
AAV	Adeno-associated virus
DMF	Dimethyl fumarate
PROTAC	Proteolysis-targeting chimera
siRNA	Small interfering RNA
NAC	N-acetylcysteine
BBB	Blood–brain barrier
NBP	dl-3-n-butylphthalide
GLP-1	Glucagon-like peptide-1
GWAS	Genome-wide association study
PRS	Polygenic risk score
PPMI	Parkinson’s Progression Markers Initiative
CSF	Cerebrospinal fluid
MRI	Magnetic resonance imaging
NSAIDs	Non-steroidal anti-inflammatory drugs
SASP	Senescence-associated secretory phenotype
Aβ	Amyloid-beta
IGF-1	Insulin-like growth factor-1
EETs	Epoxyeicosatrienoic acids
GRIN2A	Glutamate ionotropic receptor NMDA type subunit 2A
CYP1A2	Cytochrome P450 family 1 subfamily A member 2
*MAPT*	Microtubule-associated protein tau
